# Evaluation of antigen detection and polymerase chain reaction for diagnosis of amoebic liver abscess in patients on anti-amoebic treatment

**DOI:** 10.1186/1756-0500-5-416

**Published:** 2012-08-07

**Authors:** Virendra Jaiswal, Ujjala Ghoshal, Sanjay S Baijal, Balraj Mittal, Tapan N Dhole, Uday C Ghoshal

**Affiliations:** 1 Department of Microbiology, Sanjay Gandhi Postgraduate Institute of Medical Sciences, Lucknow 226014, India; 2 Department of Radiology, Sanjay Gandhi Postgraduate Institute of Medical Sciences, Lucknow 226014, India; 3 Department of Genetics, Sanjay Gandhi Postgraduate Institute of Medical Sciences, Lucknow 226014, India; 4 Department of Gastroenterology, Sanjay Gandhi Postgraduate Institute of Medical Sciences, Lucknow 226014, India

**Keywords:** *Entamoeba histolytica*, Amoebiasis, Pyogenic liver abscess, Anti-amoebic IgG antibody

## Abstract

**Background:**

Diagnosis of amoebic liver abscess (ALA) in patients on anti-amoebic drugs is difficult. There is scanty data on this issue using *Entamoeba histolytica* (*E. histolytica*) lectin antigen and polymerase chain reaction (PCR). We studied utility of lectin antigen, PCR, and IgG antibody in diagnosis of liver abscess in patients on anti-amoebic treatment. Liver aspirate of 200 patients, of which 170 had anti-amoebic drug prior to drainage, was tested for *E. histolytica* lectin antigen by (ELISA), PCR, bacterial culture, and serum IgG antibody by (ELISA). Classification of abscesses was based on result of anti-amoebic IgG antibody and bacterial culture, *E. histolytica* PCR and bacterial culture, and *E. histolytica* lectin antigen and bacterial culture.

**Findings:**

Using anti-amoebic IgG antibody and bacterial culture, 136/200 (68.0%) were classified as ALA, 12/200 (6.0%) as pyogenic liver abscess (PLA), 29/200 (14.5%) as mixed infection, and 23/200 (11.5%) remained unclassified. Using amoebic PCR and bacterial culture 151/200 (75.5%) were classified as ALA, 25/200 (12.5%) as PLA, 16/200 (8.0%) as mixed infection, and 8/200 (4.0%) remained unclassified. With *E. histolytica* lectin antigen and bacterial culture, 22/200 (11.0%) patients were classified as ALA, 39/200 (19.5%) as PLA, 2/200 (1.0%) as mixed infection, and 137/200 (68.5%) remained unclassified.

**Conclusions:**

*E. histolytica* lectin antigen was not suitable for classification of ALA patients who had prior anti-amoebic treatment. However, PCR may be used as alternative test to anti-amoebic antibody in diagnosis of ALA.

## Findings

### Introduction

Amoebic liver abscess (ALA) is caused by protozoan parasite *Entamoeba histolytica* (*E. histolytica*), a common parasitic infection in tropical countries [1-3]. Approximately 50 million people are infected with *E. histolytica* annually world-wide, with mortality ranging from 40,000 to 1,000,00 [4]. Most of the mortality due to amoebiasis results from hepatic rather than intestinal infection. Clinical and radiological features of ALA are often somewhat similar with pyogenic liver abscess (PLA). Hence, necessitating laboratory investigation for differentiation between ALA and PLA are required [5]. Currently ALA is distinguished from PLA by microscopic examination, anti-amoebic IgG serology, and culture of aspirate for pyogenic organisms.

Detection of trophozoites on microscopic examination in liver aspirate, though confirmatory of ALA, is quite insensitive [6,7]. Diagnosis of ALA is most frequently made using serum anti-amoebic IgG antibody [8,9]. However, this assay may not distinguish past from current infection, especially in endemic regions [10]. Thus, anti-amoebic IgG antibody may also be detected in a proportion of healthy people [10,11].

Sensitivity and specificity of *E. histolytica* lectin antigen have been reported to be as high as 80-90% in stool and serum specimens for diagnosis of amoebiasis [12-14]. Detection of *E. histolytica* lectin antigen in liver aspirate may also be useful for diagnosis of liver abscesses. Unfortunately, scanty data available on lectin antigen detection in liver aspirate are often contradictory [14,15]. Several PCR types like conventional, nested, touchdown and real time have been developed that detect *E. histolytica* DNA in stool samples and liver aspirate [7,12,16-21]. Sensitivity and specificity of conventional PCR is comparable with touchdown as well as real time PCR [20,22]. Recently, amoebic DNA has been reported in saliva and urine specimens with low sensitivity [23,24]. *E. histolytica* DNA detection in liver aspirate has been found to be promising for diagnosis of ALA in a few recent studies on a small number of patients [14,19,25,26]. Since the administration of antibiotics and anti-amoebic drugs is common in developing countries like India where amoebiasis is endemic, it is essential to evaluate various diagnostic tests in these patients. Accordingly, we aimed to evaluate the anti-amoebic IgG antibody test, *E. histolytica* lectin antigen assay and conventional PCR in association with bacterial culture for the diagnosis of patients with liver abscess most of whom were on anti-amoebic treatment.

### Methods

We examined 220 patients who were subjected to drainage of liver abscesses in the radiology department of a tertiary care center over a 3-yr-period (January 2006 to December 2009). Among 200 patients with well-defined liver abscess, 170 (85.0%) received an anti-amoebic drug prior to drainage. Liver abscesses were diagnosed by abdominal ultra-sonography. Patients not requiring aspiration, or drainage, or finally diagnosed to have tubercular or fungal abscesses were excluded from the study. Demographic and clinical parameters were recorded in a standard questionnaire. Informed consent was obtained from all the patients and the protocol was approved by the Institution’s (Sanjay Gandhi Postgraduate Institute of Medical Sciences, Lucknow-India) Ethics Committee (PGI/DIR/RC/957/2007).

Five ml of liver aspirate obtained during drainage of abscesses were examined for bacteria using Gram staining and bacterial culture was also done using standard media [27]. Sera obtained from these patients were stored at -40 ° C till tested. Anti-amoebic IgG antibody was assayed using a commercially available kit (Nova Tec Immunodiagnostica GmbH, CITY, Germany) following the manufacturer’s instructions. India is endemic for amoebiasis. Accordingly, 100 age and sex matched volunteers were used as healthy controls for determination of anti-amoebic IgG antibody titer in healthy population. Based on optical density (OD) results from healthy people, a cut-off OD value was determined using receiver operating characteristic (ROC) curve. Serum with an absorbance in excess of cut-off OD on 450 nm was considered positive. *E. histolytica* lectin antigen in liver aspirate was identified using commercially available kit (TechLab *E. histolytica* II, Blacksburg, Virginia) within 24 hr or stored at -40 ° C for no more than 7 days.

DNA was isolated directly from the liver aspirate samples using CTAB (hexadecyltrimethylammonium bromide) method [28]. A 125 base pair region of extra-chromosomal circular DNA of *E. histolytica* was amplified as previously described [19]. All PCR assays were repeated twice for the validation of the result. DNA extracts from *E. histolytica* strains HM-1:1MSS were used as positive controls and *E. dispar* CDC 0784 as a negative control in each PCR run.

Sensitivity, specificity, positive, and negative predictive values (PPV, NPV) were calculated using standard formulae [29]. Continuous and qualitative variables were analyzed using independent sample *t*-test, Pearson’s Chi-square, and Fisher’s exact test wherever appropriate. Two-tailed *P*-values <0.05 were considered as significant.

### Results

Twenty patients were excluded from the final analysis as a definite diagnosis could not be made due to inadequate work-up (n = 12), or drainage not needed (n = 4), tubercular liver abscesses (n = 2), and fungal liver abscesses (n = 2). Demographic, clinical, imaging and laboratory data of 200 patients with liver abscess are shown in Additional file 1: Table S1. Patients with ALA were younger in age, more often male, and had history of alcoholism, diarrhea in the recent past, or blood in the stool (dysenteric). In contrast, patients with PLA were more often diabetic, and had gall stones. On laboratory investigation, patients with PLA had lower levels of serum albumin, alanine aminotransferase (ALT), aspartate aminotransferase (AST), and higher hemoglobin than those with ALA. A solitary lesion in right lobe of liver was seen more frequently in ALA than PLA on imaging.

Bacteria were found in 41 of 200 (20.5%) liver aspirate samples on microscopic examination of the Gram stained smears and cultures. In 28 of 41 (68.3%) patients, there was a single species of bacteria in their aspirate. However, 13 of 41 (31.7%) patients had mixed infection of bacteria in their aspirate. These included *Escherichia coli* (n = 19), *Klebsiella pneumoniae* (n = 12), *Pseudomonas aeruginosa* (n = 6), *Staphylococcus* sp. (n = 4), *Acinetobacter baumannii* (n = 4), *Enetrobacter* sp. (n = 4), *Proteus vulgaris* (n = 2), *Morganella* sp. (n = 1).

Based on the OD from healthy controls, a cut-off OD value was designated. Best sensitivity (83%) and specificity (63%) of a commercially available kit was calculated using ROC curve at OD = 0.291. In total, 165 of 200 (82.5%) liver abscess patients produced an OD >0.291. However, 35 of 200 (17.5%) patients with liver abscesses had an OD < 0.291, while 141 of 170 (82.9%) patients had anti-amoebic IgG antibody in their sera, which were collected after initiation of the anti-amoebic drug. In contrast, 24 of 30 (80.0%) patients had anti-amoebic IgG antibody in their sera, which were collected before initiation of the anti-amoebic drug (*P* = ns).

*E. histolytica* lectin antigen was present in 24/200 (12%) liver aspirate. Detection of lectin antigen in liver aspirate was significantly lower in patients who had received prior anti-amoebic treatment (4/170, 2.4% vs 20/30, 66.7%, P < 0.001).

Amoebic DNA was detected in 167/200 (83.5%) patients. *E. histolytica* DNA was amplified more commonly in patients who had received prior anti-amoebic drugs (144/170, 84.7% vs 23/30, 76.7%, *P* > 0.05).

#### Classification of liver abscesses using anti-amoebic IgG antibody and bacterial culture

In 136 of 200 (68%) patients with liver abscess classified as ALA as anti-amoebic IgG antibody was present in their sera, and aspirate was sterile on bacterial culture. Twelve of 200 (6.0%) patients were classified as pyogenic as their sera did not show anti-amoebic IgG antibody, while their aspirate culture revealed growth of a pyogenic organism. Twenty-nine of 200 (14.5%) were classified as mixed infection since their sera had anti-amoebic IgG antibody and aspirate culture revealed presence of bacteria; 23 of 200 (11.5%), however, remained unclassified as their sera did not have anti-amoebic IgG antibody and aspirate was also sterile on bacterial culture (Table 1).

**Table 1:** **Classification of patients with liver abscess using various such as tests anti-amoebic IgG antibody, *E. histolytica *PCR, *E. histolytica *lectin antigen and bacterial culture (n=200)**

	**Amoebic liver abscess**	**Pygenic liver abscess**	**Mixed Infection**	**Unclassified**
IgG Ab + Bacterial Culture	136, (68%)	12, (6%)	29, (14.5%)	23, (11.5%)
Amoebic DNA + Bacterial Culture	151, (75.5%)	25, (12.5%)	16, (8%)	8, (4%)
Lectin Antigen + Bacterial Culture	22, (11%)	39, (19.5%)	2, (1%)	137, (68.5%)

#### Classification of liver abscesses using *E. histolytica* PCR and bacterial culture

In 151 of 200 (75.5%) patients with liver abscess were classified as ALA as their aspirate had *E. histolytica* DNA and bacterial culture was sterile. Twenty-five of 200 (12.5%) were classified as pyogenic, since their aspirate did not show *E. histolytica* DNA but grew bacteria on culture. Sixteen of 200 (8.0%) patients were classified as having a mixed infection as their aspirate had both *E. histolytica* DNA and bacteria grew on culture, while 8 of 200 (4.0%) remained unclassified as their aspirate did not have *E. histolytica* DNA and bacterial culture was sterile (Table 1).

#### Classification of liver abscesses using *E. histolytica* lectin antigen and bacterial culture

Twenty-two of 200 (11.0%) patients with liver abscess were classified as amoebic since their aspirate had *E. histolytica* lectin antigen, but was sterile on bacterial culture. Thirty-nine of 200 (19.5%) were classified as pyogenic as their aspirate did not show *E. histolytica* lectin antigen, but bacteria grew on culture, and 2 of 200 (1.0%) were classified as mixed infections as their aspirate was positive both for *E. histolytica* lectin antigen and bacterial culture. However, 137 of 200 (68.5%) remained undiagnosed as their aspirate was negative for both *E. histolytica* lectin antigen and bacterial culture (Table 1).

Using a combination of various tests, patients not classified into any category was highest by *E. histolytica* lectin antigen and bacterial culture and lowest using *E. histolytica* PCR and bacterial culture [8/200 (4.0%), 23/200 (11.5%), and 137/200 (68.5%) *P* = 0.000]. The number of patients classified as ALA was highest using *E. histolytica* PCR and bacterial culture, compared to anti-amoebic IgG antibody and *E. histolytica* lectin with bacterial culture [151/200 (75.5%), 136/200 (68.0%), and 22/200 (11%) *P* = 0.000] (Table 1).

#### Sensitivity and specificity

Patients with mixed and unclassified infections were excluded for the calculation of sensitivity, specificity, PPV, and NPV. Considering anti-amoebic IgG antibody and bacterial culture as gold standards, sensitivity, specificity, PPV, and NPV of *E. histolytica* PCR and *E. histolytica* lectin antigen ELISA were 99%, 91%, 99%, 91% and 15%, 100%, 100%, 9.3%, respectively. Using *E. histolytica* PCR and bacterial culture as gold standard, sensitivity, specificity, PPV, and NPV of anti-amoebic IgG antibody and *E. histolytica* lectin antigen ELISA were 89%, 56%, 90%, 44% and 14.5%, 100%, 100%, 17%, respectively. The measurement of agreement between anti-amoebic IgG antibody and *E. histolytica* lectin antigen was low [kappa value (k) = 0.002] and association between these 2 tests were also not significant (*P* = 0.911). However, anti-amoebic IgG antibody and *E. histolytica* PCR had agreement (k = 0.456) and the 2 tests were significant (*P* < 0.001, Table 2).

**Table 2:** Agreement between anti-amoebic IgG antibody with *E. histolytica *-lectin antigen and *E. histolytica *-PCR among ALA* and unclassified patients (n=159)

		**Disease status by IgG**		**Total**	**Kappa value**	**P value**
		**ALA**	**Unclassified**			
Disease status by *E. histolytica* Ag	ALA	20	2	22	0.020	0.447
	Unclassified	116	21	137		
Total		136	23	159		
Disease status	ALA	135	15	150	0.456	0.001
by PCR	Unclassified	1	8	9		
Total		136	23	159		

*Amoebic liver abscess

### Discussion

The current study showed that *E. histolytica* lectin antigen in association with bacterial culture of liver aspirate was less useful in classifying liver abscesses. In contrast, serum anti-amoebic IgG antibody or *E. histolytica* PCR in combination with bacterial culture classified most of the liver abscesses and confirmed the biological significance of these conditions to a reasonable degree [3].

Our study showed that TechLab *E. histolytica* lectin antigen test was not useful for diagnosis of ALA. This is in contrast to 2 previously reported results in which sensitivity of this test in liver aspirate sample were 40.7% and 50.0%, respectively [14,15]. Anti-amoebic drugs are known to cause false negative results using *E. histolytica* lectin antigen assay in an animal model [30]. In a study published recently from Malaysia, 29 of 32 (90.6%) patients with ALA [31], *E. histolytica* lectin antigen had become negative in sera within 48 hr of metronidazole administration. Another study showed, 3 of 7 (42.9%) patients were positive for *E. histolytica* lectin antigen prior to treatment while only 3/40 (7.5%) patients had lectin antigen during or after treatment [14]. The same study also reported that 9/11 (81.8%) serum samples of patients with ALA had become negative for *E. histolytica* lectin antigen after 1 wk of anti-amoebic treatment. In developing countries where amoebiasis is endemic, anti-amoebic drugs are often used indiscriminately. This could explain the low sensitivity of *E. histolytica* lectin antigen test on liver aspirate in our study. Thus, *E. histolytica* lectin antigen test is not useful for diagnosis of ALA in patients receiving anti-amoebic treatment.

One sample was negative by PCR but positive by IgG antibody. The possible explanation to this could be presence of PCR inhibitors in liver aspirate as present in stool samples. Although, we used inhibitors tablet to optimize the PCR, some PCR inhibitors might be co-extracted with the DNA, which inhibited the PCR.

One interesting finding of the present study is 2 patients with of ALA, which were diagnosed by PCR but not by IgG antibody. Presence of amoebic DNA in two liver aspirates specimens, establishes amoebic infection in them. The negative result of ELISA (IgG) shows that the host made an alternate mechanism for defense against the parasite. The above cases present an interesting area of research on defense mechanism in host infected with *E. histolytica*.

In the present study we have used conventional PCR for diagnosis of ALA. Using *E. histolytica* PCR with bacterial culture, most of patients with liver abscess were classified as ALA. *E. histolytica* PCR had greater sensitivity for diagnosis of ALA than anti-amoebic IgG antibody. Twenty-three patients with liver abscess in the present study could not be unclassified using anti-amoebic antibody with bacterial culture. However, only 8 patients were remained unclassified by *E. histolytica* PCR with bacterial culture. Thus in country like India where real time PCR is not available in all laboratories, conventional PCR can be used for the diagnosis of ALA. It reduces cost of sample processing compared to in real time and nested PCR.

### Conclusions

*E. histolytica* lectin antigen in combination with bacterial culture was least useful in classifying liver abscesses in patients who had received anti-amoebic drug prior to collection of the liver aspirate sample; in contrast, a combination of serum anti-amoebic antibody or *E. histolytica* PCR and bacterial culture classified most of the liver abscesses successfully.

## Abbreviations

ALA: Amoebic liver abscess; PLA: Pyogenic liver abscess; PCR: Polymerase chain reaction; *E. histolytica*, *Entamoeba histolytica*; ALT: Alanine aminotransferase; AST: Aspartate aminotransferase; OD: Optical density; ROC: Receiver operating characteristics.

## Competing interests

The authors declare that they have no financial and non financial competing interests.

## Authors’ contribution

UG-Additional Professor and UCG- Additional Professor conceived the study; BM-Professor and TND-Professor designed the protocol; SSB-Professor drained liver pus from patients; VJ-PhD student carried out microscopy, ELISA, PCR; VJ, UG and UCG analysed the data and drafted manuscript; UCG performed the statistical analysis; all authors read and approved the final version. UG is guarantor of the paper.

## Supplementary Material

Additional file 1 **Table S1. **Demographic and clinical features of 200 liver abscess patients classify using anti-amoebic IgG antibody and bacterial culture. Click here for file
