# Supplementary material for: Evaluation of antigen detection and polymerase chain reaction for diagnosis of amoebic liver abscess in patients on anti-amoebic treatment
Source: BMC Res Notes. 2012 Aug 7;5:416. doi: 10.1186/1756-0500-5-416 (PMC3477060; doi:10.1186/1756-0500-5-416)
Supplement: Additional file 1 — Table S1. Demographic and clinical features of 200 liver abscess patients classify using anti-amoebic IgG antibody and bacterial culture. [file 1756-0500-5-416-S1.docx]

Table 1: Demographic and clinical features of 200 liver abscess patients classify using anti-amoebic IgG antibody and bacterial culture.

| **Parameters** | **ALA**  **(n=136, 68%)** | **PLA**  **(n=12, 6%)** | **Mixed infection (n=29, 14.5%)** | **Unclassified**  **(n=23, 11.5%)** | **p value** |
| --- | --- | --- | --- | --- | --- |
| Male | 122 (89%) | 7 (58%) | 28 (96%) | 16 (69%) | 0.001 |
| Age | 41.1±15.6 | 42.2±23.1 | 44.3±12.9 | 45.8±15.7 | NS |
| **History** |  |  |  |  |  |
| Alcoholism | 59 (43%) | 1 (8%) | 3 (10%) | 7 (30%) | 0.001 |
| Diabetes mellitus | 8 (6%) | 4 (33%) | 9 (31%) | 6 (26%) | <0.001 |
| Gall stone | 3 (2%) | 7 (58%) | 9 (31%) | 2 (8%) | <0.001 |
| History of prior diarrhoea | 63 (46%) | 6 (50%) | 13 (45%) | 17 (74%) | <0.001 |
| Blood in stool | 24 (18%) | 6 (50%) | 5 (17%) | 3 (13%) | 0.04 |
| **Sign symtomps** |  |  |  |  |  |
| Fever | 117 (86%) | 6 (50%) | 14 (48%) | 19 (82%) | <0.001 |
| Edema | 103 (76%) | 3 (25%) | 4 (14%) | 4 (17%) | <0.001 |
| Jaundice | 29 (21%) | 2 (17%) | 16 (55%) | 2 (8%) | <0.001 |
| **Lab parameter** |  |  |  |  |  |
| ESR (mm 1^st^ per/h) † | 63.14 | 75.92 | 79.0 | 88 | 0.002 |
| TLC (1000/μl) † | 13.6 | 19.9 | 15.8 | 17.2 | <0.001 |
| Albumin (g/dl) † | 2.9 | 2.2 | 3.08 | 3.50 | 0.004 |
| Total bilurubin (mg/dl) † | 1.64 | 3.30 | 1.61 | 1.27 | <0.001 |
| ALT (U/L) † | 55.37 | 40.5 | 35.17 | 46.09 | 0.007 |
| ALP (U/L) † | 254.2 | 571 | 393 | 385 | <0.001 |
| Total protein (g/dl) † | 5.66 | 5.13 | 5.98 | 5.16 | NS |
| Serum Creatinine (mg/dl) † | 2.27 | 1.69 | 2.24 | 2.13 | NS |
| AST (U/L) † | 72.2 | 60.05 | 49.3 | 50.5 | 0.04 |
| Hb % (g/dl) † | 11.5 | 13.02 | 12.6 | 9.9 | 0.005 |
| **Imaging** |  |  |  |  |  |
| Solitary | 120 (88%) | 6 (50%) | 14 (48%) | 19 (82%) | 0.001 |
| Right lobe | 113 (83%) | 6 (50%) | 12 (41%) | 17 (73%) | 0.001 |
| Left lobe | 8 (6%) | 1 (8%) | 2 (7%) | 2 (8%) | NS |
| Both lobe | 15 (11%) | 5 (41%) | 15 (52%) | 4 (17%) | 0.001 |
| Anchovy sauce pus | 125 (92%) | 0 | 6 (20%) | 19 (82%) | 0.001 |
| Yellow pus | 11 (8%) | 12 (100%) | 23 (79%) | 4 (17%) | 0.001 |

† Mean values, NS= not significant, ESR, Erythrocyte sedimentation rate; TLC, Total leukocyte count; ALT, alanine amino transferase; ALP, alkaline phophatase, AST, aspartate aminotransferase
